# Synergistic effects of floral phytochemicals against a bumble bee parasite

**DOI:** 10.1002/ece3.2794

**Published:** 2017-02-15

**Authors:** Evan C. Palmer‐Young, Ben M. Sadd, Rebecca E. Irwin, Lynn S. Adler

**Affiliations:** ^1^ Department of BiologyUniversity of Massachusetts at AmherstAmherstMAUSA; ^2^ School of Biological SciencesIllinois State UniversityNormalILUSA; ^3^ Department of Applied EcologyNorth Carolina State UniversityRaleighNCUSA

**Keywords:** antimicrobial synergy, bumblebee, *Crithidia bombi*, plant secondary metabolites, pollinator–parasite interactions, trypanosome

## Abstract

Floral landscapes comprise diverse phytochemical combinations. Individual phytochemicals in floral nectar and pollen can reduce infection in bees and directly inhibit trypanosome parasites. However, gut parasites of generalist pollinators, which consume nectar and pollen from many plant species, are exposed to phytochemical combinations. Interactions between phytochemicals could augment or decrease effects of single compounds on parasites. Using a matrix of 36 phytochemical treatment combinations, we assessed the combined effects of two floral phytochemicals, eugenol and thymol, against four strains of the bumblebee gut trypanosome *Crithidia bombi*. Eugenol and thymol had synergistic effects against *C. bombi* growth across seven independent experiments, showing that the phytochemical combination can disproportionately inhibit parasites. The strength of synergistic effects varied across strains and experiments. Thus, the antiparasitic effects of individual compounds will depend on both the presence of other phytochemicals and parasite strain identity. The presence of synergistic phytochemical combinations could augment the antiparasitic activity of individual compounds for pollinators in diverse floral landscapes.

## 1 Introduction

Plant communities comprise species that produce distinct and varied combinations of phytochemicals (Hartmann, 1996). Floral phytochemicals, including those found in nectar and pollen, play a variety of ecological roles, including acting as antimicrobials that protect plants and their flowers against pathogens (Huang et al., 2012; Junker & Tholl, 2013; McArt, Koch, Irwin, & Adler, 2014). Phytochemical combinations can have effects that differ from predictions based on activities of isolated components. In the incremental evolution of phytochemical‐based defenses in plants, new phytochemicals would be selected for activity in the context of a plant's preexisting phytochemical repertoire, rather than for functional value in isolation (Richards et al., 2016). Plants can therefore be expected to contain chemical components that, in addition to providing protection from diverse antagonists, act to potentiate each other's activities, and thereby economize resource allocation to defensive chemicals. However, even in well‐established areas of chemical ecology such as plant–herbivore interactions, surprisingly few studies have explicitly examined the interacting effects of chemicals in mixtures (Richards et al., 2016), leaving much to be understood regarding the ecological functions of phytochemical mixtures and diversity.

In addition to defending plants against their own pathogens, antimicrobial phytochemicals can also counteract infection in animals, including pollinators (Karban & English‐Loeb, 1997; de Roode, Lefèvre, Hunter, Lefevre, & Hunter, 2013; Singer, Mace, & Bernays, 2009). Medicinal effects of phytochemicals are especially relevant for bees, given that bees have abundant access to phytochemicals in nectar and pollen and that some species are threatened by parasite‐related population decline (Cameron et al., 2011; Goulson, Nicholls, Botías, & Rotheray, 2015). Several studies have shown that individual floral phytochemicals can reduce parasite infections in bees. High concentrations of thymol (100 ppm) reduced *Nosema ceranae* infection in honey bees (Costa, Lodesani, & Maistrello, 2010); realistic nectar concentrations of gelsemine (Manson, Otterstatter, & Thomson, 2010), four of eight other floral phytochemicals (Richardson et al., 2015) reduced *Crithidia bombi* parasitism in *Bombus impatiens,* and naturally occurring concentrations of nicotine ameliorated *C. bombi* infection in *B. terrestris* (Baracchi, Brown, & Chittka, 2015). In addition, eugenol and thymol had direct inhibitory effects on *C. bombi* growth, with inhibitory concentrations of thymol (4.5–22 ppm) close to those measured in floral nectar (5.2–8.2 ppm) (Palmer‐Young et al. in press).

In nature, pollinators and their parasites encounter phytochemicals in combination rather than individually. Many bees are generalist pollinators that forage from a variety of plants. For example, in grasslands, a single bumblebee species may forage on as many as 13 plant species (Goulson & Darvill, 2004). Moreover, phytochemical combinations occur within individual plants. For example, more than 60 compounds were present in floral essential oils of *Helichrysum arenarium* (Lemberkovics et al., 2001), 37 compounds were identified from *Thymus zygus* (Pina‐Vaz et al., 2004), and over 100 compounds were found in the nectar of *Epipactis helleborine* (Jakubska, Przado, Steininger, Aniolł‐Kwiatkowska, & Kadej, 2005). Pollen is similarly rich in phytochemicals (Dobson & Bergstrom, 2000; Ketkar et al., 2014). Nectar‐derived honey also has abundant floral phytochemicals (Viñas, Soler‐Romera, & Hernández‐Córdoba, 2006), with 147 compounds identified from eight types of monofloral honey; these honeys inhibited pro‐ and eukaryotic pathogens, including *Staphylococcus aureus, Escherichia coli*, and *Candida albicans* (Isidorov, Bagan, Bakier, & Swiecicka, 2015).

Functional interactions among chemicals fall into three general categories: additive, antagonistic, and synergistic effects (Jia et al., 2009). Additive effects indicate that the effects of chemicals are independent of one another. This can occur when the chemicals have similar modes of action, such that adding a second compound has the same effect as adding more of the first compound (Greco, Bravo, & Parsons, 1995), or when the two compounds target independent processes that have minimal effects on one another (Tallarida, 2000). A clinical example of additive effects due to independent actions would be the activities of two phytochemicals, artemisinin and curcumin, against malaria (Nandakumar, Nagaraj, Vathsala, Rangarajan, & Padmanaban, 2006). Artemisinin interferes with mitochondrial function (Krishna, Woodrow, Staines, Haynes, & Mercereau‐Puijalon, 2006), while curcumin causes DNA damage (Cui, Miao, & Cui, 2007). Assessments of interactions between compounds often compare results observed to results predicted under a null hypothesis of additivity (Greco et al., 1995).

Antagonistic effects occur when two compounds inhibit one another's activities, such that mixtures are less effective than predicted based on the activities of each compound in isolation. At the extreme, one compound is an antidote to a compound known to cause toxicity. Antagonistic effects can occur, for example, when one compound alters a structure that is a target of a second compound, or interferes with the production of a second compound's target (Jia et al., 2009). Other mechanisms may include reduced uptake or stimulation of detoxification (Gershenzon & Dudareva, 2007). An example of antagonistic effects is the coprecipitation of tomato leaf saponins and phytosterols. Although each can be toxic in isolation, binding between saponins and phytosterols reduces absorption and bioavailability of both compounds (Duffey & Stout, 1996).

Synergistic effects occur when two compounds increase one another's potency, resulting in mixtures that have stronger effects than predicted based on activities of their components in isolation. Synergistic effects are especially useful in clinical situations. By reducing the dose required to achieve a medicinal effect, selectively synergistic drug combinations can both reduce costs and lower the risk of patient toxicity (Greco et al., 1995). Plants, which have evolved to produce defensive mixtures under conditions of limited resources and diverse antagonists, are an intuitive place to look for synergistic chemical combinations (Richards et al., 2016). Generally speaking, synergy can occur when one compound increases the bioavailability (Smith, Roddick, & Jones, 2001), inhibits the detoxification (Berenbaum & Neal, 1985), or compromises the export of another compound (Stermitz, Lorenz, Tawara, Zenewicz, & Lewis, 2000).

Functional interactions between co‐occurring phytochemicals could alter how plant chemistry mediates pollinator–parasite relationships, but although several studies have tested the effects of phytochemical mixtures, few have specifically addressed interactions between multiple compounds. For example, phytochemically complex, antimicrobial resins (Simone‐Finstrom & Spivak, 2012), and certain types of honey (Gherman et al., 2014) may decrease infection in honey bees, and honey derived from multiple plant species had stronger antimicrobial properties than monofloral honey (Erler, Denner, Bobiş, Forsgren, & Moritz, 2014). However, none of these studies quantified the contributions of individual versus combined phytochemical components to the biological activity of the tested mixtures. The few studies that explicitly tested the effects of mixtures relevant to pollinators have produced results that ranged from potential synergy to antagonism. In one study*,* neither nicotine nor thymol alone affected *C. bombi* infection in *B. impatiens,* but nectar containing both compounds at low concentrations (2 ppm nicotine + 0.2 ppm thymol) tended to reduce infection intensity (Biller, Adler, Irwin, McAllister, & Palmer‐Young, 2015), suggesting that the two compounds have synergistic effects. However, resin mixtures gathered by stingless bees had additive and less than additive effects against several test microbes in vitro (Drescher, Wallace, Katouli, Massaro, & Leonhardt, 2014), and in *B. impatiens,* a nicotine–anabasine mixture lacked the medicinal value of each compound alone against *C. bombi* (Thorburn, Adler, Irwin, & Palmer‐Young, 2015), suggesting antagonistic effects.

**Figure 1:**
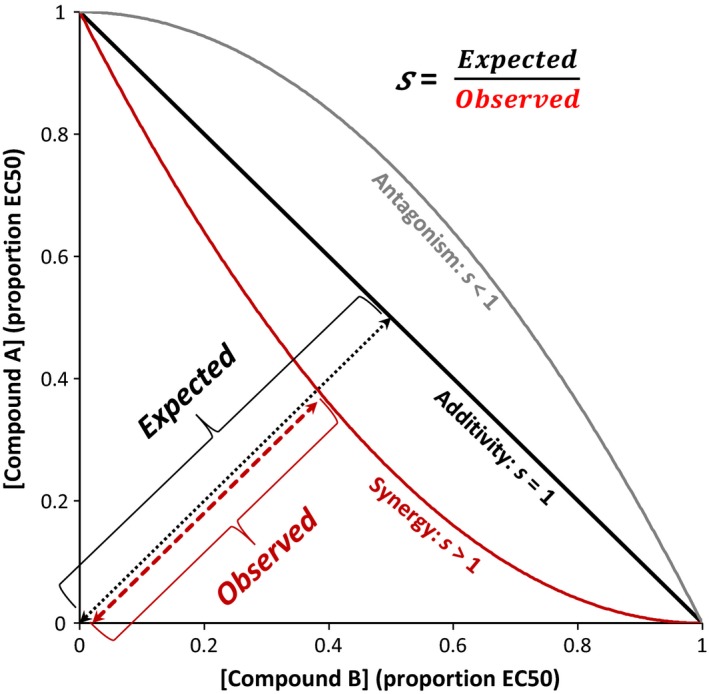
Schematic depicting the shapes of growth isoclines for different patterns of interaction. Interactions between the two compounds are quantified by the parameter *s,* which reflects the ratio of the *Expected* to *Observed* concentrations that result in 50% inhibition. The solid black line represents the shape of the growth isocline under the null hypothesis of additivity, corresponding to *s = 1*. The red parabola depicts the concave shape of the isocline when there is synergy between the two compounds (*Expected > Observed, s > 1*), whereas the gray parabola depicts a convex isocline, which occurs when the compounds have antagonistic effects (*s < 1*). For clarity, the distance *Observed* is only shown for the case of synergy

Characterization of parasite‐inhibiting interactions between multiple phytochemicals *in vitro* has the potential to link studies of single compounds with studies of complex phytochemical suites that occur in nature. We used cell cultures of the bumblebee parasite *C. bombi* to assess the individual versus combined effects of two widespread antimicrobial floral phytochemicals, eugenol and thymol, on parasite growth. Parasite cell cultures allow for efficient and high‐resolution characterization of the direct effects of individual compounds (Palmer‐Young et al. in press) and their combinations. Such approaches are commonly used for screening clinical drugs; they eliminate variation between individual hosts and allow sufficient replication to test the effects of multiple compounds across a range of doses. Using a statistical approach designed to assess the effects of two‐drug combinations (Greco et al., 1995), we mathematically defined and graphically illustrated the three classes of interaction between phytochemicals (additive, antagonistic, and synergistic, as introduced above and in Figure 1). When parasite growth isoclines are plotted for concentrations of the two chemicals, each type of interaction produces distinctively shaped isoclines: Additive interactions produce straight lines; synergistic interactions produce concave curves; and antagonistic interactions produce convex curves (Figure 1).

## 2 Study System

The trypanosome gut parasite of bumblebees, *Crithidia bombi*, potentially encounters a diverse suite of phytochemicals throughout its life cycle, making it a relevant system for addressing the effects of individual phytochemicals and combinations. *Crithidia bombi* is exposed to phytochemicals both directly at flowers, where the parasite is transmitted between hosts (Durrer & Schmid‐Hempel, 1994; Graystock, Goulson, & Hughes, 2015), and in the bee intestine, which contains phytochemicals from host‐ingested nectar and pollen (Hurst, Stevenson, & Wright, 2014). *Crithidia bombi* infects bees in many ecosystems worldwide (Cameron et al., 2011; Schmid‐Hempel, Schmid‐Hempel, Brunner, Seeman, & Allen, 2007), where phytochemical exposure will be complex and varied. The parasite's deleterious effects on infected bees (Brown, Schmid‐Hempel, & Schmid‐Hempel, 2003; Sadd & Barribeau, 2013), including threatened native species (Schmid‐Hempel et al., 2014), indicate its ecological and practical importance (Sadd & Barribeau, 2013).

Eugenol and thymol are two widespread floral chemicals to which *C. bombi* is likely to be simultaneously exposed at considerable concentrations (Table 1) when bees forage in diverse floral landscapes. Eugenol or its derivative, methyl eugenol, has been found in over 450 species from 80 plant families (Tan & Nishida, 2012), including in the flowers of over 100 species (Tan & Nishida, 2012). These numbers refer only to known occurrences; eugenol is recognized as a common volatile (Gupta, Schauvinhold, Pichersky, & Schiestl, 2014) and is likely to be present in many additional plant species that have not yet been sampled (Tan & Nishida, 2012). Plants known to contain eugenol include common crop species, such as *Cucurbita pepo* and *Ocimum selloi* (Martins, Casali, Barbosa, & Carazza, 1997), ornamentals such as *Rosa rugosa* (17–40% of anther volatiles; Dobson, Bergström, & Groth, 1990; Wu et al., 1985), and wild *Epipactis* (Jakubska et al., 2005) and *Gymnadenia* (Gupta et al., 2014) orchids. Eugenol synthase genes are also found in such common flowering plants as *Arabidopsis* spp., *Glycine max, Vitis vinifera, Populus* spp., *Betula* spp., *Petunia hybrida,* and *Clarkia breweri* (Gupta et al., 2014). Eugenol's presence is most extensively documented among plants of the Lamiaceae (38 species) (Tan & Nishida, 2012), which includes widely cultivated thymol‐containing herbs such as *Thymus vulgaris*,* Origanum vulgare*,* O. majorana*, and *O. dictamnus* (Daferera, Ziogas, & Polissiou, 2000). In at least four Lamiaceae species (Table 1), eugenol is found together with either thymol or thymol's isomer, carvacrol: *T. vulgaris* (Lee, Umano, Shibamoto, & Lee, 2005), *Ocimum basilicum* (Lee et al., 2005; Politeo, Jukic, & Milos, 2007), *Origanum vulgare* (Milos, Mastelic, & Jerkovic, 2000), and *O. majorana* (Deans & Svoboda, 1990). Thymol, eugenol, and carvacrol all co‐occur in inflorescences of the European *Helichrysum arenarium* (Lemberkovics et al., 2001), and eugenol has been found with the thymol isomer carvacrol in honey, although at low concentrations (<1 ppm) (Alissandrakis, Tarantilis, Pappas, Harizanis, & Polissiou, 2009) that could reflect phytochemical evaporation during storage. In addition to the documented presence of these specific compounds, the biochemical pathways that produce eugenol and thymol give rise to many structurally similar compounds that may have similar individual and interactive effects. Eugenol is produced via the shikimate pathway, and as a phenylpropene, it belongs to the second most diverse class of plant volatiles (Pichersky, Noel, & Dudareva, 2006). Thymol is produced from isoprenoid precursors via the methylerythritol phosphate (MEP) pathway from substrates involved in primary metabolism (Pichersky et al., 2006), meaning that precursors of thymol and related compounds are found in all plant species. As a terpenoid, thymol is a member of the most diverse class of plant volatiles (Pichersky et al., 2006).

**Table 1:** Published concentrations of eugenol and thymol in selected plants. Concentrations are given in ppm fresh mass when possible. Where references quantified concentrations in percent of essential oil per unit dry mass, concentrations were converted based on other studies that quantified leaf moisture content and/or essential oil yield, as explained in “Notes” column

Species	Sample type	Concentration	References	Notes
A. Plant species high in eugenol				
*Ocimum selloi*	Leaves	~1,200 ppm	Martins et al. (1997)	0.2% essential oil by fresh mass, 63% eugenol in oil
	Flowers	~2,400 ppm	Martins et al. (1997)	0.4% essential oil by fresh mass, 63% eugenol in oil
*Ocimum basilicum*	Leaves (broad‐leaf variety)	~70 ppm	Wogiatzi, Papachatzis, Kalorizou, Chouliara and Chouliaras (2011)	500 ppm in dried leaves; 86% leaf moisture (Rocha, Lebert, & Marty‐Audouin, 1993). *O. basilicum* may also contain thymol (Lee et al., 2005)
	Leaves (narrow‐leaf variety)	~100 ppm	Wogiatzi et al. (2011)	700 ppm in dried leaves; 86% moisture (Rocha et al., 1993)
*Rosa x hybrida*	Stamens	50 ppm	Bergougnoux et al. (2007)	13.1% of 380.6 ppm total analytes
*Cucurbita pepo* cv. Tosca	Petals	0.99–1.2 ppm	Granero, Gonzalez, Sanz, and Vidal (2005)	
	Nectar	0.02–0.57 ppm	Granero et al. (2005)	
*Dianthus caryophyllus*	Floral volatiles	Trace‐84.1% of emissions	Clery, Owen, Chambers and Thornton‐Wood (1999)	
*Gymnadenia densiflora*	Flower headspace	0.839 ppm	Gupta et al. (2014)	
*Rosmarinus* spp.	Monofloral honey	0.02–0.03 ppm	Castro‐Vázquez, Pérez‐Coello and Cabezudo (2003)	
B. Plant species high in thymol				
*Lippida sidoides*	Leaves	~8,200	de Medeiros et al. (2011)	1.06% oil in leaves (Veras et al., 2012), 78% thymol in oil
*Origanum dictamnus*	Leaves	~1,300	Daferera et al. (2000)	1.05% essential oil by mass (Argyropoulou, Papadatou, Grigoriadou, Maloupa, & Skaltsa, 2014), 78% thymol in oil, 84% moisture in leaves (Loghmanieh, Bakhoda, & Issa, 2013).
*Origanum vulgare*	Leaves and flowers	~990 ppm	De Martino, De Feo, Formisano, Mignola and Senatore (2009)	2.3% essential oil by dry mass. 63% thymol in oil, 84% moisture in leaves (Loghmanieh, Bakhoda, and Issa, 2013). *O. vulgaris* may also contain eugenol (De Martino et al., 2009; Milos et al., 2000)
*Thymus vulgaris*	Leaves	~,3200 ppm	Daferera et al. (2000)	~0.5% essential oil by fresh mass (Hudaib, Speroni, Di Pietra, & Cavrini, 2002), 64% thymol in oil
*Thymus vulgaris*	Leaves	~1,370 ppm	Lee et al. (2005)	8550 ppm in dried leaves; assume 84% moisture in leaves (Loghmanieh et al., 2013). *T. vulgaris* may also contain eugenol (Lee et al., 2005)
*Thymus pulegioides* L.	Leaves and flowers	~1,500 ppm	Senatore (1996)	0.5% essential oil by fresh mass, 30% thymol in oil
*Satureja montana*	Leaves	~1,000 ppm	Nikolić et al. (2014)	1.5% essential oil by dry mass (Sefidkon, Jamzad, & Mirza, 2004), 44% thymol in oil, 84% moisture in leaves (Loghmanieh, Bakhoda, and Issa, 2013)
*Origanum majorana*	Leaves	~1,100 ppm	Daferera et al. (2000)	Assume 0.5% essential oil by fresh mass (Hudaib et al., 2002), 14% thymol in oil. *O. majorana* may also contain eugenol (Deans & Svoboda, 1990)
*Thymus vulgaris*	Nectar	5.2–8.2 ppm	Palmer‐Young, Sadd et al. (2016)	
*Thymus* spp.	Honey	0.27 ppm	Nozal, Bernal, Jiménez, González and Higes (2002)	

Both eugenol and thymol have recognized antitrypanosomal effects, including against *C. bombi* (Palmer‐Young, Sadd, Stevenson, Irwin, & Adler, 2016), with 50% growth inhibition of *Trypanosoma cruzi* by 76–246 ppm eugenol and 53–62 ppm thymol (Santoro, Cardoso, Guimarães, Mendonça, & Soares, 2007; Santoro, Cardoso, Guimarães, Salgado et al., 2007). Combinations of thymol and eugenol had synergistic effects against *Escherichia coli* (Pei, Zhou, Ji, & Xu, 2009), but antagonistic effects against *Crithidia fasciculata* (Azeredo & Soares, 2013). However, compounds with similar or overlapping targets typically have additive effects (Jia et al., 2009). Eugenol and thymol are similar in chemical structure—each is a lipophilic compound with an aromatic ring and free hydroxyl group; eugenol and thymol also had similar effects on cell morphology of *Trypanosoma cruzi* (Santoro, Cardoso, Guimarães, Mendonça et al., 2007; Santoro, Cardoso, Guimarães, Salgado et al., 2007). Therefore, we predicted that eugenol and thymol would have additive effects on *C. bombi*.

## 3 Materials and Methods

Seven independent experiments were conducted with four *C. bombi* strains. The first six experiments were conducted on strains tested singly *in series*, with three rounds of experiments on strain IL13.2 and one experiment each on strains VT1, C1.1, and S08. To account for week‐to‐week differences between experimental conditions, the final experiment tested all four strains *in parallel*, i.e., strains were tested concurrently, but with reduced replication of treatments within strains.

### 3.1 Parasite culturing

Parasite strains were isolated from wild bumblebees collected near Normal, IL, USA, in 2013 (“IL13.2,” from *B. impatiens*, collected by BMS); Hanover, NH, USA, in 2014 (“VT1,” from *B. impatiens,* by lab of REI); Corsica, France, in 2012 (“C1.1,” from *B. terrestris*, collected by BMS); and Zurich, Switzerland, in 2008 (“S08,” from *B. terrestris*, collected by the group of Paul Schmid‐Hempel, which included BMS).

Strains were isolated by flow cytometry‐based single‐cell sorting of bee feces (IL13.2, C1.1, S08) or homogenized intestinal tracts (strain VT1) as described previously (Salathé, Tognazzo, Schmid‐Hempel, & Schmid‐Hempel, 2012). All strains were isolated directly from wild bees with the exception of VT1, which was first used to infect laboratory colonies of *B. impatiens* (provided by Biobest, Leamington, ON, Canada). The cell used to initiate the parasite culture was obtained from an infected worker of one of the commercial colonies. Cultures were microscopically screened to identify samples with strong *Crithidia* growth and the absence of bacterial or fungal contaminants and then stored at −80°C in a 2:1 ratio of cell culture:50% glycerol until several weeks before the experiments began. Thereafter, strains were incubated in tissue culture flasks at 27°C. Strains were propagated twice per week at a density of 100 cells/μl in 5‐ml fresh culture medium, the composition of which has been previously described (Salathé et al., 2012). The final transfer (to 500 cells/μl in 5‐ml fresh medium) occurred 48 h before the experiment began.

### 3.2 Experimental design

Eugenol (Acros, Thermo Fisher, Franklin, MA, USA) and thymol (Fisher Scientific, Franklin, MA, USA) treatment media were prepared by predissolving phytochemicals in ethanol to 40 mg/ml; ethanol solutions were stored at −20°C. Phytochemicals were then dissolved in growth media to create two stock solutions at 4× desired concentrations, one of eugenol (800 ppm in IL13.2, Rounds 1 & 2; 1,600 ppm for all other experiments with strains tested in series; 1,200 ppm for strains tested in parallel) and another of thymol (200 ppm in IL13.2, Rounds 1 & 2; 400 ppm in other in‐series experiments; 300 ppm for strains tested in parallel). Six twofold dilutions of this stock were made separately for each phytochemical. Ethanol was added to treatments of lesser concentrations to equalize the ethanol concentrations (2–4% v/v for eugenol and 0.5–1% v/v for thymol, depending on the experiment) in all treatments. A fully crossed phytochemical treatment matrix consisting of all 36 possible combinations at 2× their desired final concentrations was prepared in a 2‐ml deep‐well 96‐well plate, with eugenol treatments in rows and thymol treatments in columns. Using a multichannel pipette, we transferred 100 μl 2× treatment media to the inner 36 wells of six (for experiments in series) or two (for strains tested in parallel) replicate 96‐well tissue culture‐treated plates. Hence, each plate contained a single well at each of the 36 two‐phytochemical treatment combinations, and each experiment included either two (for experiments in series) or six (for strains tested in parallel) biological replicates at each concentration. The treatment concentrations were chosen with the goal of achieving complete growth inhibition at the highest concentrations, in order to allow construction of dose–response curves without the need for extrapolation of inhibitory effects beyond the tested concentration range (see Section 3.3). These concentrations (0–400 ppm eugenol, 0–100 ppm thymol) spanned the range of known nectar and pollen phytochemical concentrations, but were less than maximal leaf concentrations of eugenol and thymol (Table 1).

Immediately before the assay, parasite cells from tissue culture flasks were diluted to a density of 1,000 cells/μl in 6 ml of culture medium. Cells (100 μl) were added to an equal volume of the 2× phytochemical treatment media using a multichannel pipette, thereby diluting the cells to 500 cells/μl and phytochemicals to the desired concentrations (1× with 0.625–1.25% v/v ethanol). Two additional plates were seeded with cell‐free medium rather than cells; these plates served as negative controls. Sterile distilled water was added to the outer wells of all plates to reduce evaporation and edge effects.

Plates were sealed with laboratory film and incubated inside zippered plastic sandwich bags for 5 days at 27°C. For the experiment with strain S08 tested “in series,” an additional day of growth measurements was included in the model due to slow growth over the first 5 days. Growth was measured by OD (optical density) readings (630 nm) at 24‐hr intervals. Two techniques were used before each reading to ensure accurate OD measurements: First, cells were resuspended (40 s, 1,000 rpm, 3 mm orbit) using a microplate shaker before each reading. Second, to minimize error due to condensation, the cover of the assay plate was briefly switched with that of an empty, sterile plate under sterile conditions. We calculated net OD (i.e., the amount of OD resulting from parasite growth) by subtracting the average OD reading from cell‐free control wells of the corresponding phytochemical treatment and time point.

### 3.3 Statistical analysis

All statistical analyses were carried out using the open‐source software R v3.2.1 (R Core Team 2014). We used the R package grofit (Kahm, Hasenbrink, Lichtenberg‐Fraté, Ludwig, & Kschischo, 2010) to fit a model‐free spline to the observed OD measurements. This spline fit was used to compute each sample's 5‐day growth integral (i.e., area under the curve of net OD vs. time). This growth integral was used as the response variable in subsequent analyses.

The effects of the individual phytochemicals and their interaction were assessed with a seven‐parameter Universal Response Surface Analysis as described by Greco *et al*. (1990), Greco et al. (1995). This method, which provides a statistical estimate of the interactions between compounds, has been deemed both robust and accurate for assessment of drug combinations (Meletiadis, Verweij, Dorsthorst, Meis, & Mouton, 2005; Zhao, Au, & Wientjes, 2010) and has been used in previous two‐compound studies (e.g., Faessel, Slocum, Rustum, & Greco, 1999; Greco, Park, & Rustum, 1990). The following equations were used:

(1) $$g\left(c\right)\;=\;\frac{\left(g_{\max}-g_{\min}\right){\left(\frac{c}{EC_{50}}\right)}^{m}}{1+{\left(\frac{c}{EC_{50}}\right)}^{m}}+g_{\min}$$

(2) $$\begin{array}{cc} 1\;=\; & \frac{c_{1}}{EC_{50\left(1\right)}{\left(\frac{g_{c1,c2}-g_{min}}{g_{max}-g_{c1,c2}}\right)}^{\frac{1}{m_{1}}}}+\frac{c_{2}}{EC_{50\left(2\right)}{\left(\frac{g_{c1,c2}-g_{min}}{g_{max}-g_{c1,c2}}\right)}^{\frac{1}{m_{2}}}}\\ & +\frac{fc_{1}c_{2}}{EC_{50\left(1\right)}EC_{50\left(2\right)}{\left(\frac{g_{c1,c2}-g_{min}}{g_{max}-g_{c1,c2}}\right)}^{\left(\frac{1}{2m_{1}}+\frac{1}{2m_{2}}\right)}} \end{array}$$

Equation (1) describes a sigmoidal doseesponse curve in the presence of a single inhibitory compound. On the left side of the equation, “*g(c)*” indicates the amount of growth (“*g*”) as a function of phytochemical concentration (“*c*”). Parameter “*g*_max_” represents the upper limit of growth in the absence of phytochemicals; “*g*_min_” represents the lower asymptote of the curve as phytochemical concentration approaches infinity. The “*EC*_50_” (“effective concentration”) is the phytochemical concentration at which 50% of maximal growth inhibition is achieved. Parameter “*m*” describes the slope of the dose–response curve at the *EC*_50_ concentration.

Equation (2) extends the single‐compound model in equation (1) to describe the interactive effects of two phytochemicals, which are denoted with subscripts. The parameter “*f*” classifies the type of interaction between the two phytochemicals as synergy (*f *> 0), additivity (*f *= 0), or antagonism (*f *< 0). This parameter is equivalent to the interaction term of a general linear model, in which a significant interaction indicates that the effect of one factor depends on the level of another factor (Greco et al., 1995). In our case, the factors are the two phytochemicals.

Equation (2) parameters “*c*_1_” and “*c*_*2*_” represent the respective concentrations of the two phytochemicals, and “*g*_*c1,c2*_” predicts the amount of growth at a given combination of “*c*_*1*_” and “*c*_*2*_.” The parameters “*EC*_*50*_” and “*m*” are derived by fitting dose–response curves for each individual phytochemical in the absence of the other compound using equation (1). “*EC*_*50(1)*_” and “*EC*_*50(2)*_” represent the respective 50% inhibitory concentrations of each phytochemical in the absence of the other compound; and “*m*_*1*_” and “*m*_*2*_” describe how fast growth decreases at the EC50 concentration of each phytochemical in the absence of the other compound. Parameter “*g*_*min*_” denotes the lower limit of growth as phytochemical concentrations go to infinity. The units divide out of each term in the equation: Within the denominator, the growth parameters divide out and the exponent *“m”* has no units; the units also divide out for the concentration parameters in each term's numerator and denominator.

A separate model was fit for each strain and experiment round; models were fit by the “ursa” function in package “drc” (Ritz, Baty, Streibig, & Gerhard, 2015). Results were graphed in R v3.2.1 (R Core Team, 2014) packages “plot3D” (Soetaert, 2016) and “ggplot2” (Wickham, 2009).

Because the scale of the interaction parameter *f* has a nonlinear relationship to the relative activity of compounds in mixture versus in isolation, the original interaction parameter *f* was converted to the linear interaction parameter *s* (Figure 1), which quantifies the curvature in the growth isoclines (Greco et al., 1990), by solving the equation:

(3) $$f\;=\;4(s^{2}-s)$$

Here, *f* is the parameter derived from equation (2), and *s* indicates the ratio of the expected to observed concentrations that result in 50% growth inhibition (Figure 1). For example, an *s* value of 1 indicates that compounds have additive effects. In contrast, an *s* value of 2 indicates that the compounds have twice the expected inhibitory activity when in mixture, such that only half of the expected concentrations are sufficient for 50% growth inhibition.

## 4 Results

**Figure 2:**
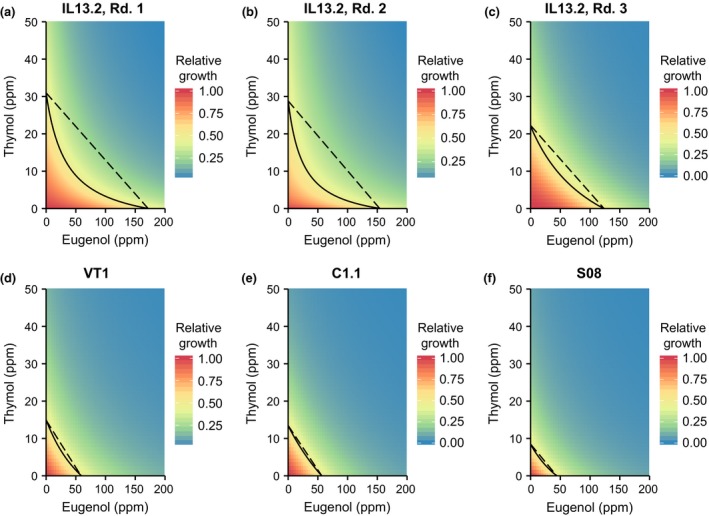
Combinatorial effects of eugenol and thymol against *C. bombi* strains tested in series over six experiments. Panels show the results of six separate experiments in separate weeks: three with *C. bombi* strain IL13.2—referred to as “Rounds 1–3,” and one each with strains VT1, C1.1, and S08. The solid line shows the isocline of 50% growth inhibition. The dashed line that connects thymol EC50 (*y*‐intercept) and eugenol EC50 (*x*‐intercept) represents the expected growth isocline if the compounds have additive effects. Concave isoclines indicate synergistic effects (see Figure 1). The plot area is color‐coded according to the predicted growth at any given vector of concentrations, with red indicating highest growth and blue indicating least growth. Growth was measured as the 5‐day growth integral, i.e., area under the curve of net OD versus time. Within each panel, growth is scaled relative to growth in the absence of phytochemicals, such that maximal growth is always equal to 1. For absolute growth measurements, refer to Figure 4d: Maximum growth. Each experiment included *n* = 216 samples (six replicate wells at each of 36 combinations of eugenol and thymol). Rd.: round. ppm: parts per million

**Figure 3:**
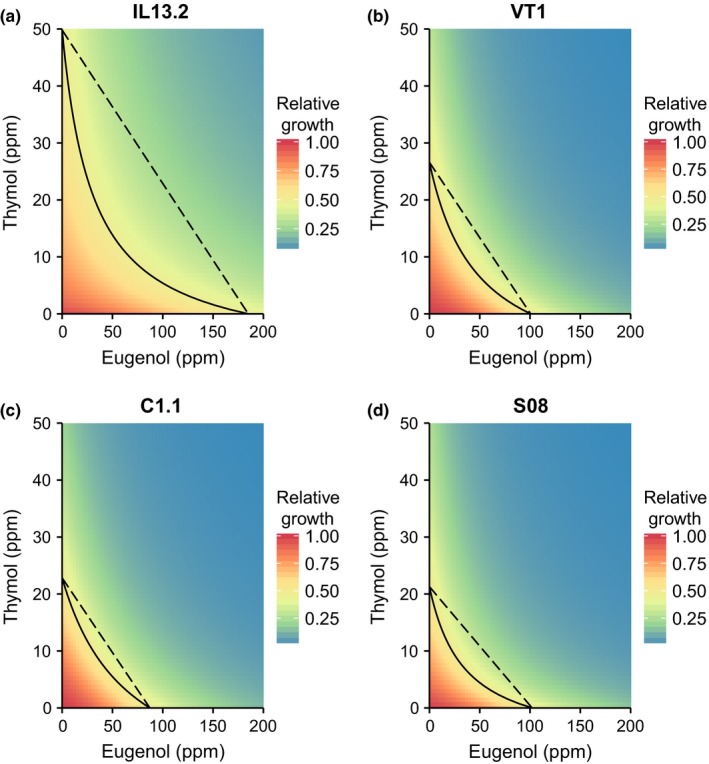
Combinatorial effects of eugenol and thymol against four *C. bombi* strains, assayed in parallel. As in Figure 2, the solid line shows the isocline of 50% growth inhibition. The dashed line that connects thymol EC50 (*y*‐intercept) and eugenol EC50 (*x*‐intercept) represents the expected growth isocline if the compounds have additive effects. Concave isoclines indicate synergistic effects (see Figure 1). Tests of each strain included *n* = 72 samples (two replicate wells at each of 36 combinations of eugenol and thymol). ppm: parts per million

**Figure 4:**
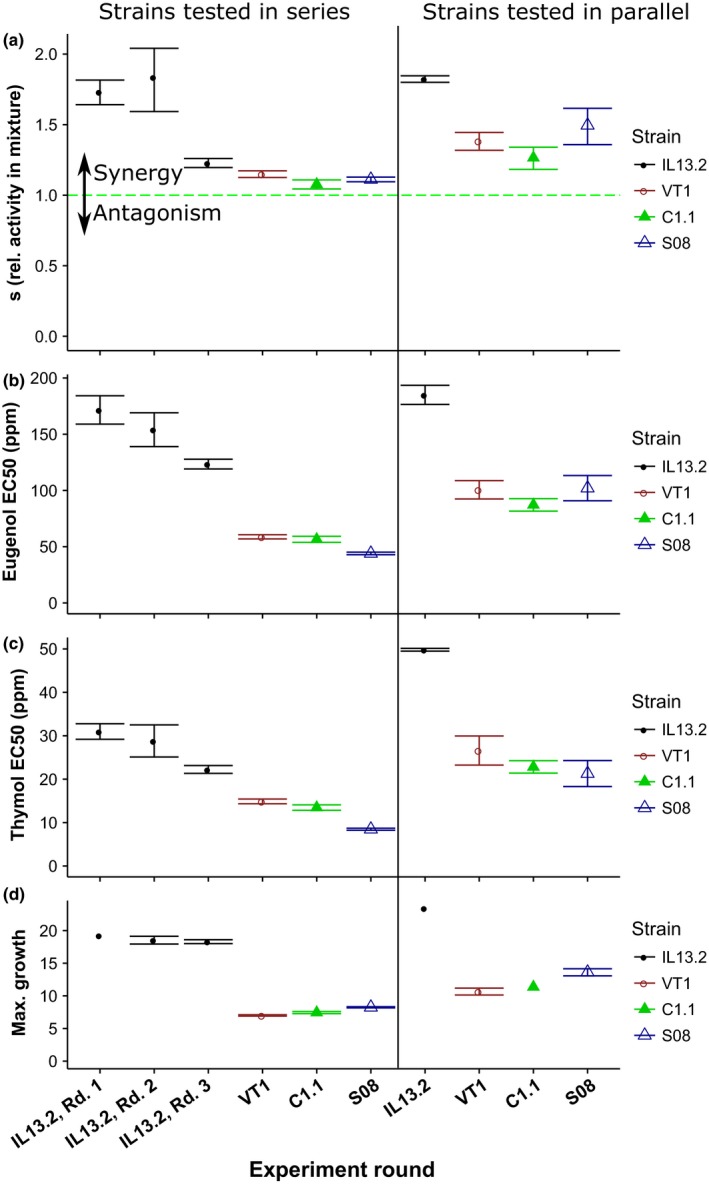
Universal Response Surface Analysis model parameters across all experiments. The *y*‐axis shows the round of the experiment. The first six experiments were conducted on strains tested singly in series, with three experiments on strain IL13.2 (“Rounds 1–3”) and one experiment each on strains VT1, C1.1, and S08. The final four experiments were conducted on all four strains tested in parallel, i.e., strains were tested concurrently. The vertical line divides the experiments conducted in series from the experiments conducted in parallel. The *x*‐axis shows model estimates and 95% CIs for four parameters: (a) *s* is the interaction parameter from equation (3), which indicates the relative potency of each compound in mixture versus in isolation. Values *s > 1* indicate synergy. The null hypothesis of additivity is indicated by the dashed green line. (b) Eugenol and (c) thymol EC50s are the individual phytochemical concentrations necessary for 50% growth inhibition. (d) Max. growth shows growth in the absence of phytochemicals, i.e., at a concentration of 0 ppm. The legend indicates color coding of points and confidence intervals by strain. Where no error bars are shown for maximum growth, this parameter was fixed as the average of growth in control samples exposed to 0 ppm phytochemicals

Eugenol and thymol had synergistic effects on the growth inhibition of *C. bombi* in each of the ten analyses, as evidenced by the shape of the growth contour lines (Figures 2 and 3) and values of the interaction parameter “*s*” (Figure 4; *s* >1 indicates synergy). The highly concave contour lines in strain IL13.2 (Figure 2a–c, Table 1) indicate that synergistic effects were most pronounced against this strain. The increase in potency due to co‐occurrence of the compounds in IL13.2 varied from 23% in Round 3 to 84% in Round 2, with statistically significant synergy in all strains and experimental rounds (Figure 4, Supplementary Table S1). Synergistic interactions were weaker but still statistically significant in strains VT1 (15% and 38% potentiation in series and in parallel, respectively), C1.1 (8% and 27%), and S08 (11% and 50%, Figure 4; see Supplementary Table S1 for full model parameters). In general, the in‐series experiments with VT1, C1.1, and S08 were characterized by poor growth, with low levels of synergy, phytochemical tolerance, and maximum growth in the absence of phytochemicals. When strains were tested in parallel, all strains grew strongly, with higher EC50 values, but also more apparent synergistic effects of the combined phytochemicals (Figure 4). The relative strength of synergy in the four strains was reasonably consistent across the in‐series and in‐parallel experiments. In both the in‐series and the in‐parallel experiments, synergistic effects were strongest against strain IL13.2, weakest against C1.1, and intermediate against VT1 and S08.

## 5 Discussion

The existence and nature of combinatorial interactions will determine how phytochemical blends can mediate plants’ interactions with mutualists, antagonists, and their diseases—including pollinator infections—in nature, where exposure to compound combinations at variable doses is inevitable. Synergistic interactions, in which chemical combinations are more effective than single components, are of particular clinical and ecological interest. Synergistic combinations can have greater efficacy against infection, or achieve medicinal effects at lower total dosage, which may reduce the risk of host toxicity (Jia et al., 2009). Our results quantitatively demonstrate how a naturally occurring phytochemical combination influences the growth of an important pollinator parasite and provide a model for future work on the role of phytochemical combinations in plant–pollinator–parasite interactions.

Eugenol and thymol exhibited synergistic inhibitory effects that varied in strength across strains and experiments. Previous work has indicated that interactions between eugenol and thymol are dependent on the focal taxon. Eugenol and thymol synergistically inhibited *E. coli* (Pei et al., 2009) and porcine gut microbiota (Michiels, Missotten, Fremaut, De Smet, & Dierick, 2007), and a eugenol–thymol–citral combination had synergistic toxicity to *Trypanosoma cruzi* (Azeredo & Soares, 2013). However, eugenol and thymol had antagonistic effects against *Crithidia fasciculata* (Azeredo & Soares, 2013). *C. bombi* is known to be genetically diverse (Salathé & Schmid‐Hempel, 2011), with genotype‐specific infection ability (Barribeau, Sadd, du Plessis, & Schmid‐Hempel, 2014) and growth rate (Ulrich & Schmid‐Hempel, 2012). Our results show that *C. bombi* strains also varied in resistance to both interphytochemical synergy and isolated phytochemicals (Palmer‐Young et al. in press). This finding has ecological importance, because, in contrast to the organisms above, *C. bombi* is naturally exposed to these phytochemicals from flowers.

The mode of action of phytochemicals can influence their interactions when in combination. Eugenol and thymol have generally similar effects against trypanosomes and other eukaryotes, although these effects can vary across taxa. Eugenol and thymol are both hydrophobic volatiles with free hydroxyl groups; they can penetrate membranes, disrupt ionic gradients needed for energy production, and precipitate oxidative stress that damages vital lipids and proteins (Bakkali, Averbeck, Averbeck, & Idaomar, 2008). In *T. cruzi,* both eugenol (Santoro, Cardoso, Guimarães, Mendonça et al., 2007) and thymol (Santoro, Cardoso, Guimarães, Salgado et al., 2007) caused cytoplasmic swelling, rounding of the cell body, and altered nuclear morphology. In *Leishmania major*, both eugenol (Ueda‐Nakamura et al., 2006) and thymol (de Medeiros et al., 2011) affected mitochondria. In the yeast *Candida albicans*, both eugenol and thymol altered membrane morphology (Braga, Sasso, Culici, & Alfieri, 2007). Although neither compound affected the plasma membrane of *T. cruzi* (Santoro, Cardoso, Guimarães, Mendonça et al., 2007; Santoro, Cardoso, Guimarães, Salgado et al., 2007), eugenol altered the mitochondrial membrane in *L. donovani* (Ueda‐Nakamura et al., 2006), and thymol caused membrane wrinkling and submembrane accumulation of lipid droplets in *L. amazonensis* (de Medeiros et al., 2011). Given the similar chemical structures and modes of action of eugenol and thymol, we predicted that these compounds would behave additively. To our surprise, eugenol and thymol had synergistic effects against all four *C. bombi* strains. Generally, compounds with synergistic effects have related but distinct cellular targets (Jia et al., 2009), rather than identical targets. Although eugenol and thymol had similar effects on trypanosome cell morphology (Azeredo & Soares, 2013), our results suggest that these compounds may have distinct complementary effects at a finer scale.

From an ecological perspective, the synergistic effects found in our study suggest that combinations of eugenol and thymol could ameliorate parasite infection in pollinators. Both eugenol and thymol are tolerated by bees at considerable concentrations. In *Apis mellifera* adults, the eugenol LD50 over 8 d was 7800 ppm (Ebert, Kevan, Bishop, Kevan, & Downer, 2007), well above the 44–185 ppm EC50 of our *C. bombi*. Similarly, the thymol LD50 of *A. mellifera* exceeded 1,000 ppm (Ebert et al., 2007), far higher than the 8.5–49.8 ppm EC50 of *C. bombi*. However, a mere 50 ppm thymol delayed *A. mellifera* larval development (Charpentier, Vidau, Ferdy, Tabart, & Vetillard, 2014) and could have similar sublethal but deleterious effects on *Bombus* spp. Synergy between the antitrypanosomal effects of co‐occurring phytochemicals could reduce the total phytochemical dose needed to ameliorate infection, thereby reducing the risk of side effects in hosts and their offspring.

Additional sampling is needed to determine the phytochemical concentrations in nectar and pollen relative to the inhibitory concentrations reported here. Although the concentrations that inhibited growth in this study were higher than those documented to date in nectar and pollen, they were well below the levels found in leaves (Table 1). Few studies have measured pollen and nectar phytochemical concentrations. Those that have reported generally lower phytochemical concentrations in nectar and pollen than in leaves (Detzel & Wink, 1993; Kessler & Halitschke, 2009), but in some cases pollen concentrations were actually higher than in leaf tissue (Frölich, Hartmann, & Ober, 2006), and were orders of magnitude higher than those in nectar (Detzel & Wink, 1993; London‐Shafir, Shafir, & Eisikowitch, 2003; Palmer‐Young, Sadd et al., 2016). Even if pollen phytochemical concentrations are less than 10% of those in leaves, such concentrations of thymol (100–820 ppm) would still be highly inhibitory (EC50 < 50 ppm). Moreover, we tested for inhibition under conditions optimized for *C. bombi* growth. In the wild, *C. bombi* is exposed to complex phytochemical blends, host immune responses (Barribeau & Schmid‐Hempel, 2013), and abiotic stresses including temperature fluctuation, osmotic stress, and desiccation (Cisarovsky & Schmid‐Hempel, 2014). Under such stressful conditions, lower concentrations might be sufficient to impede growth.

To understand the ecological importance of phytochemical combinations, future research must address not only direct effects on parasites, but also how interactions between phytochemicals are altered by host‐mediated effects. First, phytochemicals that stimulate the host immune system (Mao, Schuler, & Berenbaum, 2013), or affect intestinal muscle contraction (Tomizawa & Casida, 2003), could synergize with directly antimicrobial phytochemicals to kill or expel gut parasites. Second, if different phytochemicals are detoxified by different enzymes (Mao, Schuler, & Berenbaum, 2011), then host detoxification of a phytochemical combination might be more efficient than detoxification of a single phytochemical. As a result, gut‐dwelling parasites might experience a relatively small proportion of the ingested phytochemical combination, and parasite inhibition would require greater total ingestion of the phytochemical combination versus the single phytochemical. This result would be interpreted as antagonism between compounds. Third, although phytochemical combinations may have synergistic effects against parasites, compound combinations can also have synergistic toxic and immunosuppressive effects against insects (Berenbaum & Neal, 1985; Duffey & Stout, 1996; Richards et al., 2012), which could exacerbate the deleterious effects of floral phytochemicals on bees (Hurst et al., 2014; Nibret & Wink, 2010). Finally, insects in the wild make behavioral choices involving nonrandom collection and use of phytochemicals and may alter foraging behavior and preferences when diseased (Baracchi et al., 2015; Erler & Moritz, 2015; Karban & English‐Loeb, 1997; de Roode et al., 2013; Simone‐Finstrom & Spivak, 2012). Hence, cell culture experiments, which detect direct effects of phytochemicals, should be complemented by studies in live insects, which account for host‐mediated indirect effects.

Our quantification of the interactive effects of a phytochemical combination is a start toward integration of the effects of single chemicals with those of chemically complex ecosystems. In our experiments, interactions between two phytochemicals had synergistic inhibitory effects of varying magnitude on a pollinator parasite. Given the actual diversity of floral blends, and the possibility of additional interactions between phytochemicals and host‐mediated effects, our study alone cannot quantify the ecological significance of interactions between co‐occurring phytochemicals. Phytochemical composition of the floral community may interface with the genotypic interactions of hosts and parasites (Sadd & Barribeau, 2013) to structure patterns of infection. Further research on single and multiplant blends is needed to determine the ecological relevance of phytochemical combinations consumed by generalist and specialist pollinators, including the effects of phytochemical combinations on disease of threatened species. Because the generalist foraging habits of many pollinators result in novel phytochemical combinations, interactions between phytochemicals of similar and distinct species are equally plausible and offer immense opportunities for future investigation, from the scale of molecules to ecosystems.

## Conflict of Interest

The authors declare that there are no competing interests.

## Authors’ Contributions

BMS and ECPY conceived the study. EPY conducted the experiments, analyzed the data, and wrote the first draft of the manuscript. All authors revised the manuscript and have agreed to its submission.
