# Supplementary material for: Synergistic effects of floral phytochemicals against a bumble bee parasite
Source: Ecol Evol. 2017 Feb 15;7(6):1836–49. doi: 10.1002/ece3.2794 (PMC5355193; doi:10.1002/ece3.2794)

Supplementary Tables

Table S1. Full model parameters for the interactive effects of eugenol and thymol on four strains of *C. bombi*. Model parameters were estimated using a Universal Response Surface Analysis as described by Greco *et al.* (Greco et al. 1990, 1995). The parameter "m" describes how rapidly growth decreased at the EC50 phytochemical concentration. Parameter "gmin" represents the lower limit of growth at very high phytochemical concentrations; "gmax" represents the maximum growth in the absence of phytochemicals. The EC50 is the concentration (in ppm phytochemical) that inhibited growth by 50%. The interaction parameter "f" indicates synergy (f > 0), additivity (f = 0), or antagonism (f < 0) between the two compounds. SE: standard error. The parameter "s" is related to "f" by the equation

$$s = 4(s^2 - s)$$

where s>1 indicates synergy, s=1 indicates additivity, and s<1 indicates antagonism. Values of "NA" in the confidence interval indicate that the parameter was fixed to assist model convergence.

Strains tested in series					
				95% CI	
Round	Strain	Parameter	estimate	lower	upper
1	IL13.2	m(eugenol)	-2.10	-2.37	-1.84
1	IL13.2	m(thymol)	-1.13	-1.19	-1.07
1	IL13.2	gmin	0.50	NA	NA
1	IL13.2	gmax	19.24	NA	NA
1	IL13.2	EC50(eugenol)	171.60	158.98	184.22
1	IL13.2	EC50(thymol)	30.98	29.18	32.78
1	IL13.2	f	5.07	4.22	5.93
1	IL13.2	s	1.73	1.64	1.82

2	IL13.2	m(eugenol)	-1.73	-2.26	-1.21
2	IL13.2	m(thymol)	-0.69	-0.76	-0.61
2	IL13.2	gmin	0.53	-0.28	1.35
2	IL13.2	gmax	18.53	17.94	19.13
2	IL13.2	EC50(eugenol)	154.03	139.01	169.05
2	IL13.2	EC50(thymol)	28.81	25.11	32.51
2	IL13.2	f	6.14	3.78	8.50
2	IL13.2	s	1.84	1.59	2.04
3	IL13.2	m(eugenol)	-2.84	-3.05	-2.62
3	IL13.2	m(thymol)	-1.83	-1.93	-1.74
3	IL13.2	gmin	0.35	0.22	0.48
3	IL13.2	gmax	18.31	18.01	18.61
3	IL13.2	EC50(eugenol)	123.44	119.09	127.78
3	IL13.2	EC50(thymol)	22.24	21.34	23.14
3	IL13.2	f	1.12	0.94	1.31
3	IL13.2	s	1.23	1.20	1.26
4	VT1	m(eugenol)	-1.63	-1.71	-1.55
4	VT1	m(thymol)	-1.54	-1.62	-1.47
4	VT1	gmin	0.55	0.52	0.59
4	VT1	gmax	7.00	6.90	7.10
4	VT1	EC50(eugenol)	58.77	56.88	60.65
4	VT1	EC50(thymol)	14.88	14.34	15.43
4	VT1	f	0.69	0.57	0.81
4	VT1	s	1.15	1.13	1.17
5	C1.1	m(eugenol)	-1.93	-2.06	-1.79
5	C1.1	m(thymol)	-1.91	-2.04	-1.77
5	C1.1	gmin	0.49	0.43	0.54
5	C1.1	gmax	7.44	7.28	7.61
5	C1.1	EC50(eugenol)	56.53	53.85	59.20
5	C1.1	EC50(thymol)	13.46	12.83	14.09
5	C1.1	f	0.33	0.18	0.48
5	C1.1	s	1.08	1.04	1.11
6	S08	m(eugenol)	-1.59	-1.64	-1.54
6	S08	m(thymol)	-1.37	-1.43	-1.32
6	S08	gmin	0.46	0.44	0.49
6	S08	gmax	8.25	8.16	8.33
6	S08	EC50(eugenol)	43.99	42.87	45.11
6	S08	EC50(thymol)	8.47	8.23	8.71
6	S08	f	0.50	0.42	0.58
6	S08	s	1.11	1.10	1.13

Strains tested in parallel					
				95% CI	
Round	Strain	Parameter	estimate	lower	upper
Together	IL13.2	m(eugenol)	-1.61	-1.82	-1.39
Together	IL13.2	m(thymol)	-0.70	-0.80	-0.60
Together	IL13.2	gmin	0.50	NA	NA
Together	IL13.2	gmax	23.41	NA	NA
Together	IL13.2	EC50(eugenol)	184.99	176.48	193.51
Together	IL13.2	EC50(thymol)	49.81	49.49	50.12
Together	IL13.2	f	6.00	5.76	6.25
Together	IL13.2	s	1.82	1.80	1.85
Together	C1.1	m(eugenol)	-2.20	-2.41	-1.99
Together	C1.1	m(thymol)	-1.61	-1.74	-1.47
Together	C1.1	gmin	0.50	NA	NA
Together	C1.1	gmax	11.37	NA	NA
Together	C1.1	EC50(eugenol)	87.17	81.63	92.72
Together	C1.1	EC50(thymol)	22.82	21.39	24.25
Together	C1.1	f	1.34	0.86	1.82
Together	C1.1	s	1.27	1.18	1.34
Together	VT1	m(eugenol)	-2.46	-2.88	-2.03
Together	VT1	m(thymol)	-1.33	-1.49	-1.17
Together	VT1	gmin	0.50	NA	NA
Together	VT1	gmax	10.66	10.13	11.18
Together	VT1	EC50(eugenol)	100.60	92.43	108.76
Together	VT1	EC50(thymol)	26.60	23.25	29.95
Together	VT1	f	2.12	1.68	2.57
Together	VT1	s	1.38	1.32	1.44
Together	S08	m(eugenol)	-2.26	-2.54	-1.98
Together	S08	m(thymol)	-1.17	-1.29	-1.05
Together	S08	gmin	0.50	NA	NA
Together	S08	gmax	13.61	13.07	14.15
Together	S08	EC50(eugenol)	102.07	90.86	113.27
Together	S08	EC50(thymol)	21.29	18.30	24.28
Together	S08	f	2.96	1.95	3.98
Together	S08	s	1.50	1.36	1.62

Supplementary Figures

Supplementary Figures S1-S6: Response surface plots showing combinatorial effects of eugenol and thymol against *C. bombi* for a series of 6 experiments on 4 strains. Each graph shows the effects of the two compounds for a given strain and week. Contour lines on the basal x-y plane show isoclines of equal growth. The concave isoclines in panels indicate synergistic effects. The response surface shows model-predicted growth, points show observed growth, and drop-lines indicate the deviation between observed and predicted growth; drop-lines are not visible in some cases due to the orientation of the graph and the good fit of the model. Surfaces and points are color-coded according the z-axis, which shows the integral of growth over five days; z-axes are scaled according to each strain's maximum growth. Each experiment included n = 216 replicates (6 replicate wells at each of 36 combinations of eugenol and thymol). ppm: parts per million.

Figure S1. Strain IL13.2, Round 1

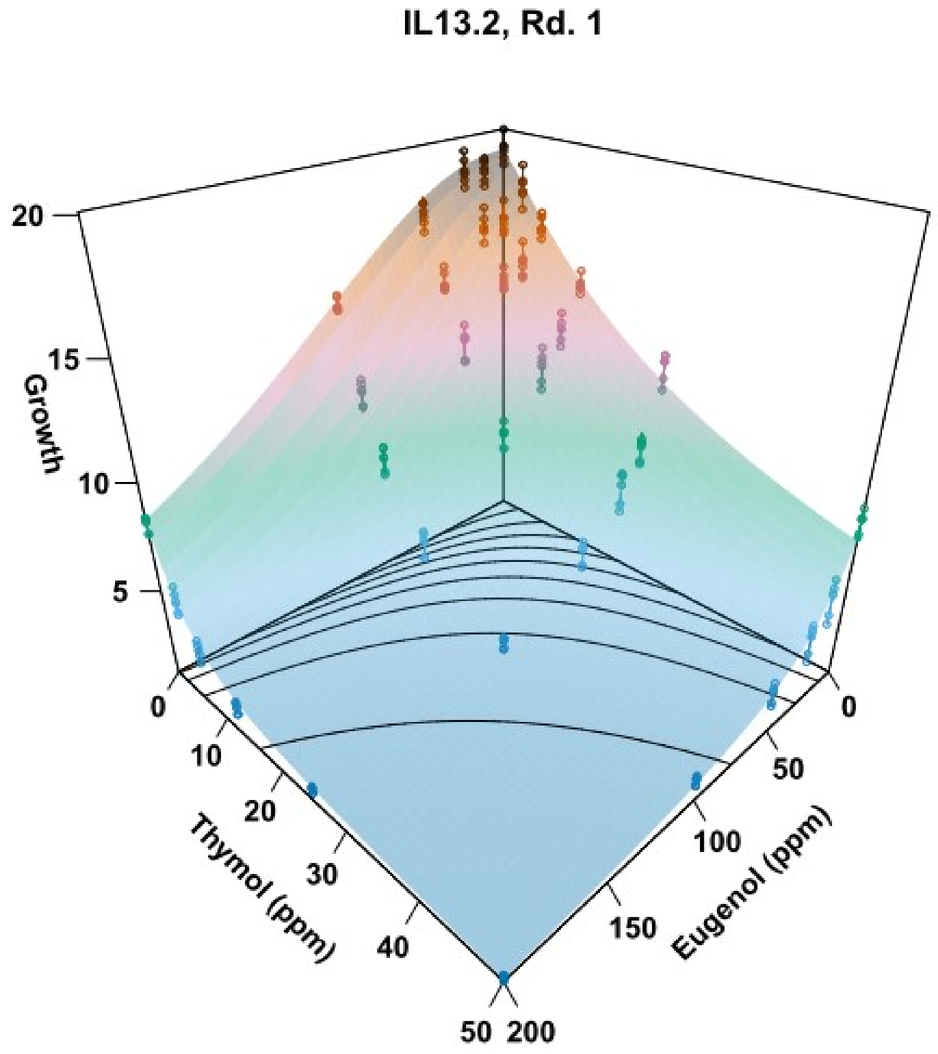

32 **Figure S2. Strain IL13.2, Round 2**

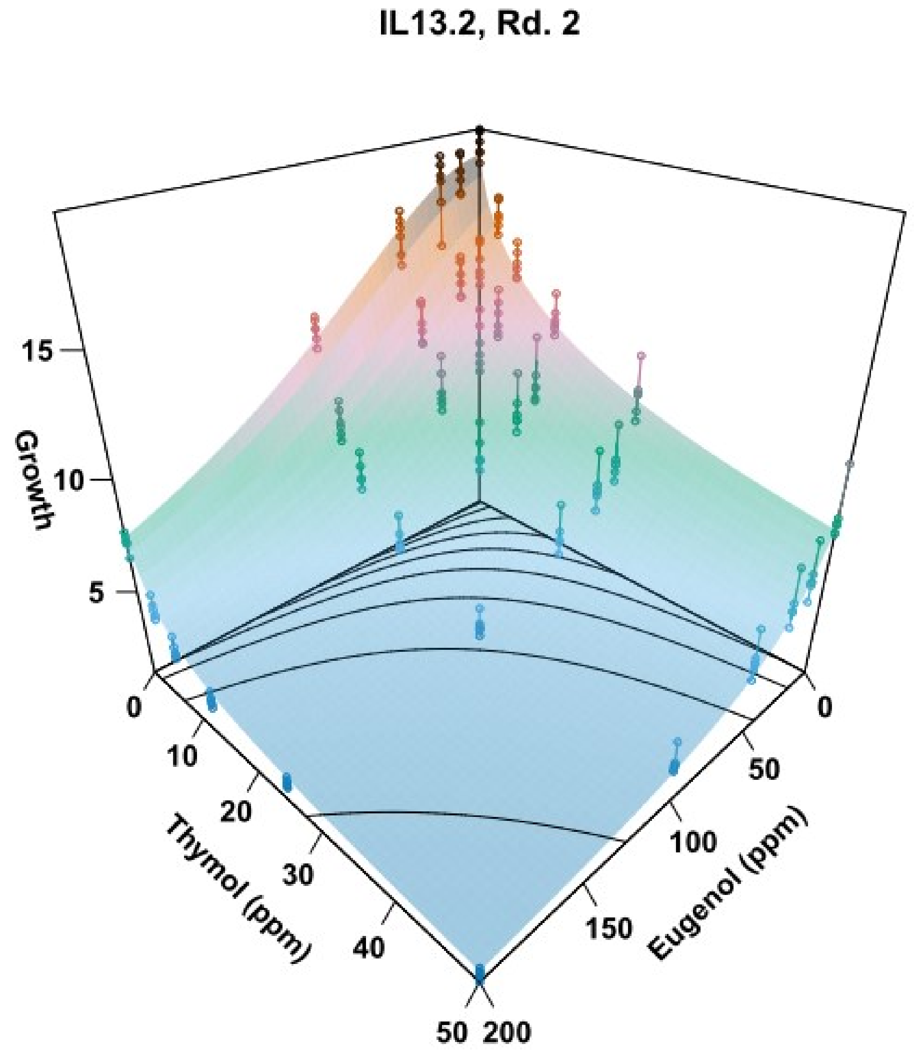

33

Figure S3. Strain IL13.2, Round 3

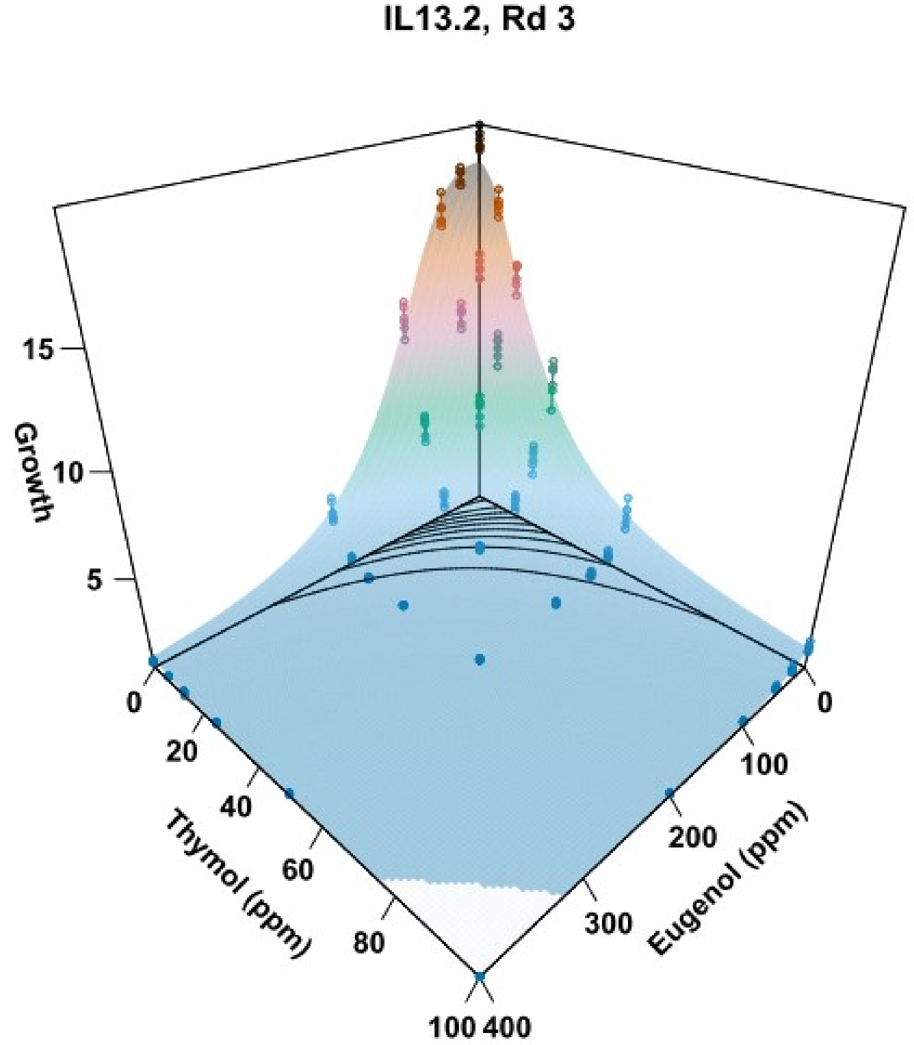

Figure S4. Strain VT1

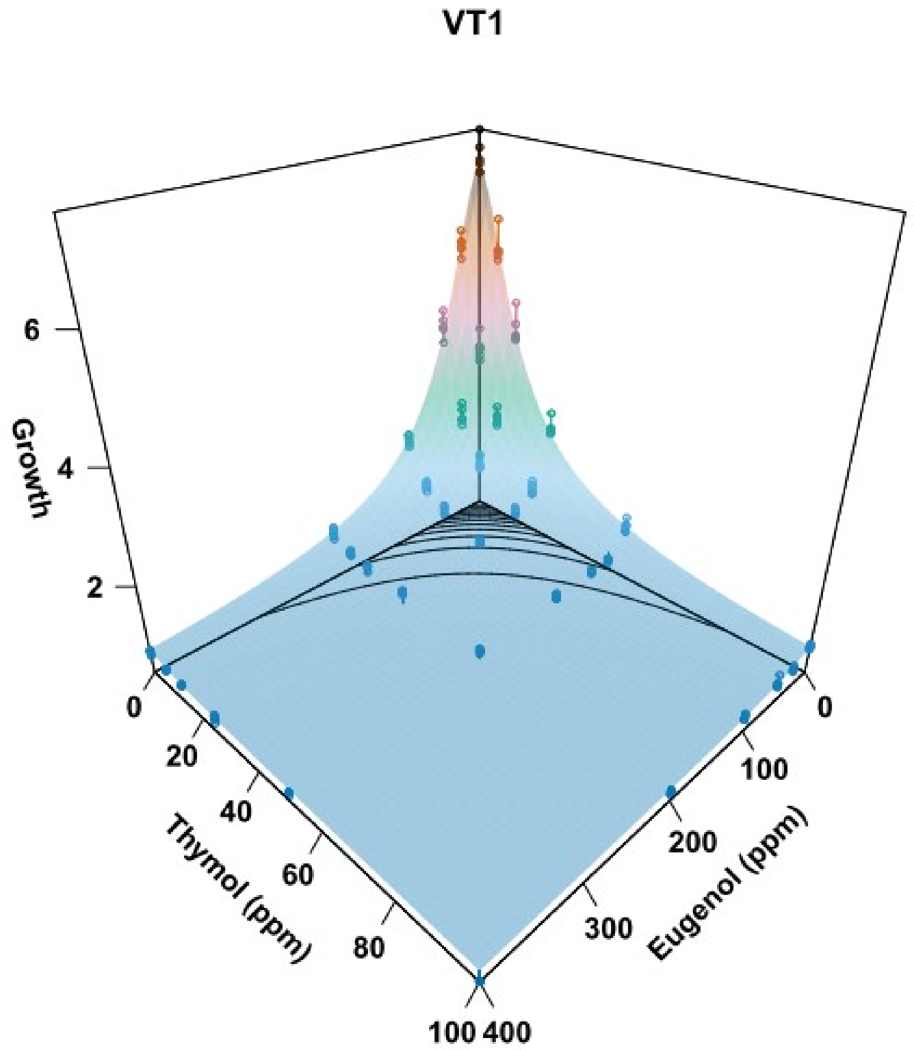

Figure S5. Strain C1.1

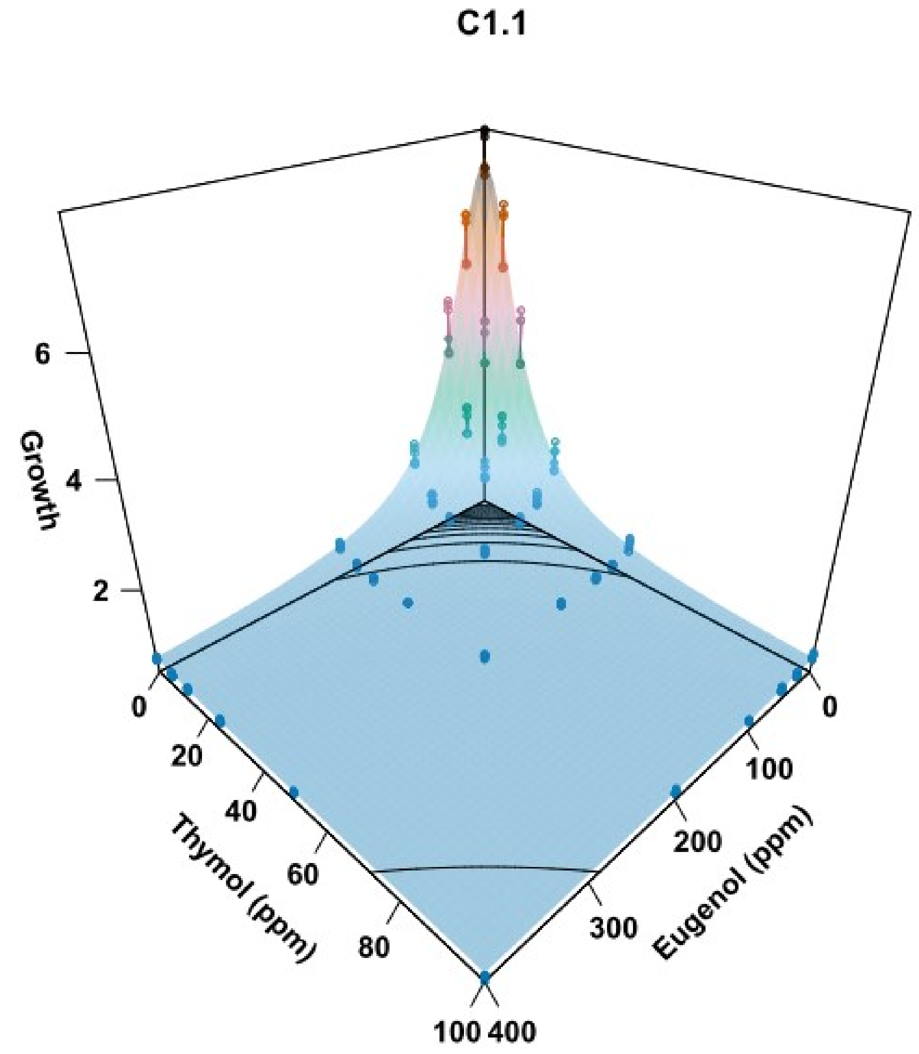

Figure S6, Strain S08

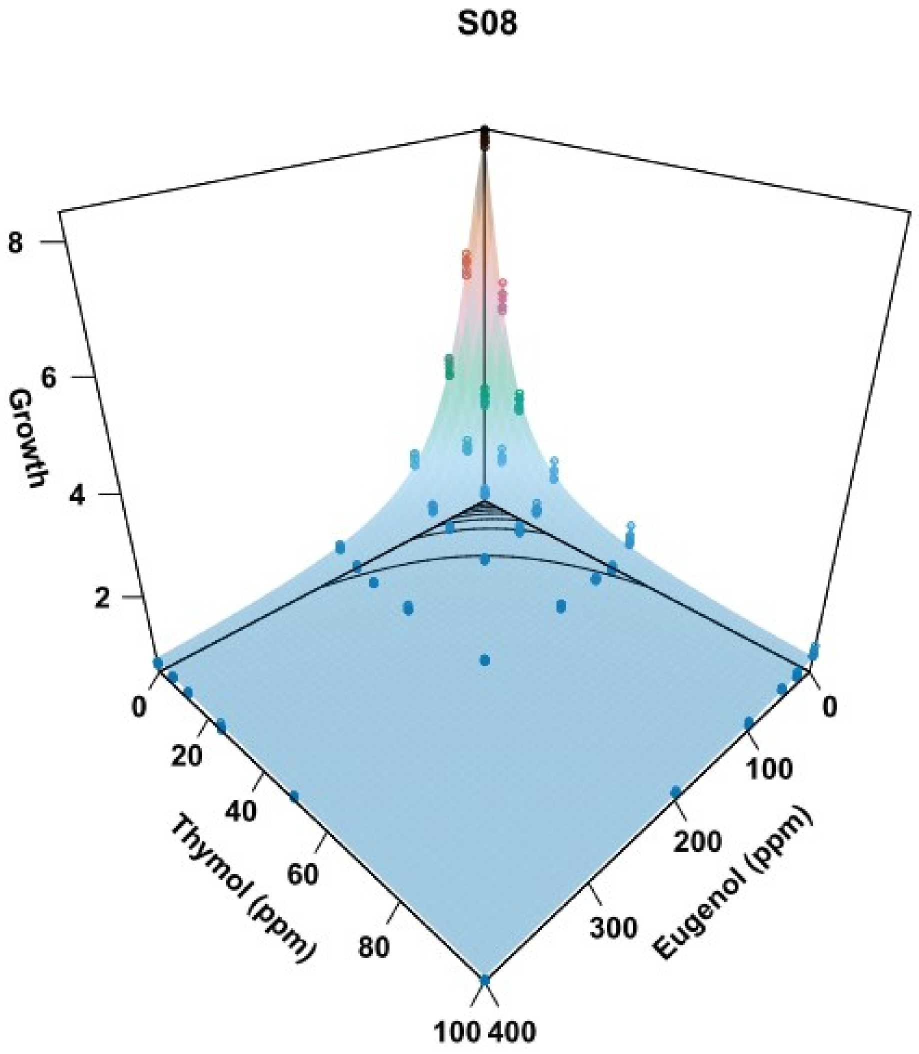

Supplementary Figures S7-S10: Response surface plots for experiments with strains tested in parallel (i.e., concurrently). Each graph shows the effects of the two compounds for a given strain and week. Contour lines on the basal x-y plane show isoclines of equal growth. The concave isoclines in panels indicate synergistic effects. The response surface shows model-predicted growth, points show observed growth, and drop-lines indicate the deviation between observed and predicted growth; drop-lines are not visible in some cases due to the orientation of the graph and the good fit of the model. Surfaces and points are color-coded according the z-axis, which shows the integral of growth over five days; z-axes are scaled according to each strain's maximum growth. Tests of each strain included n = 72 samples (2 replicate wells at each of 36 combinations of eugenol and thymol). ppm: parts per million.

53 **Figure S7. Strain IL13.2**

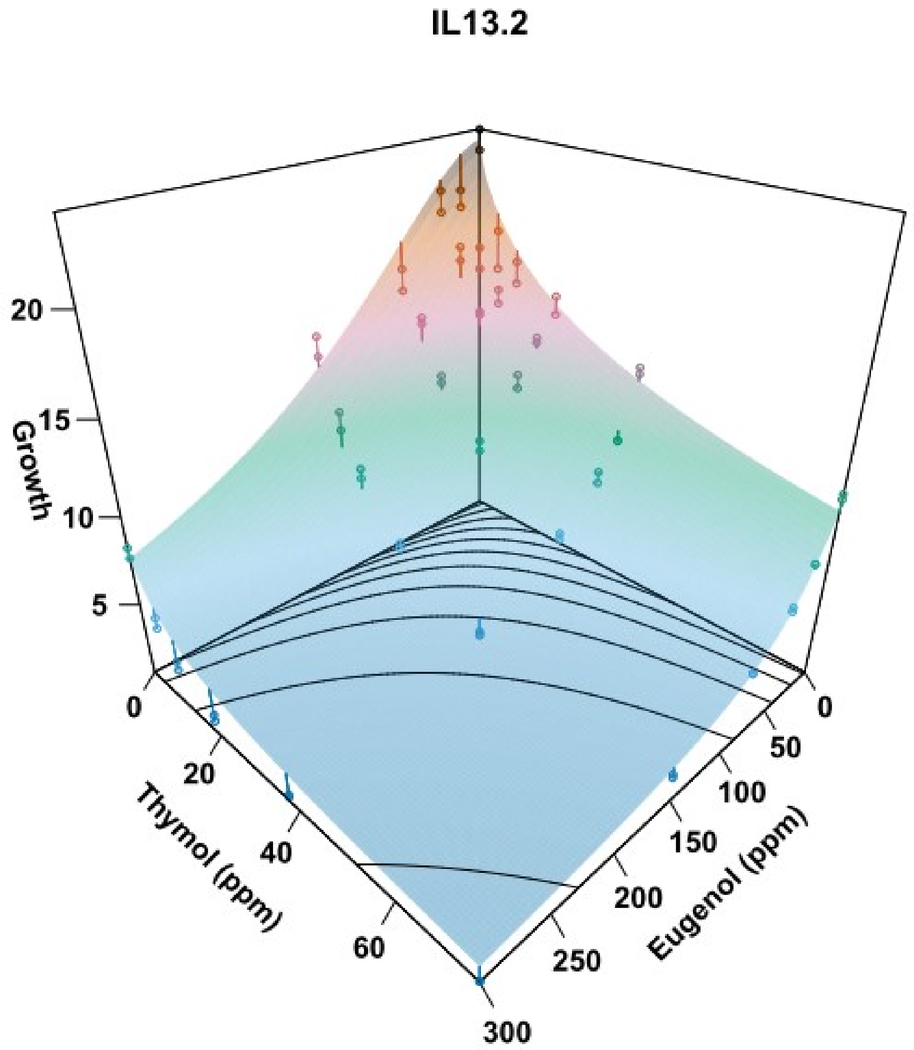

54

Figure S8. Strain VT1

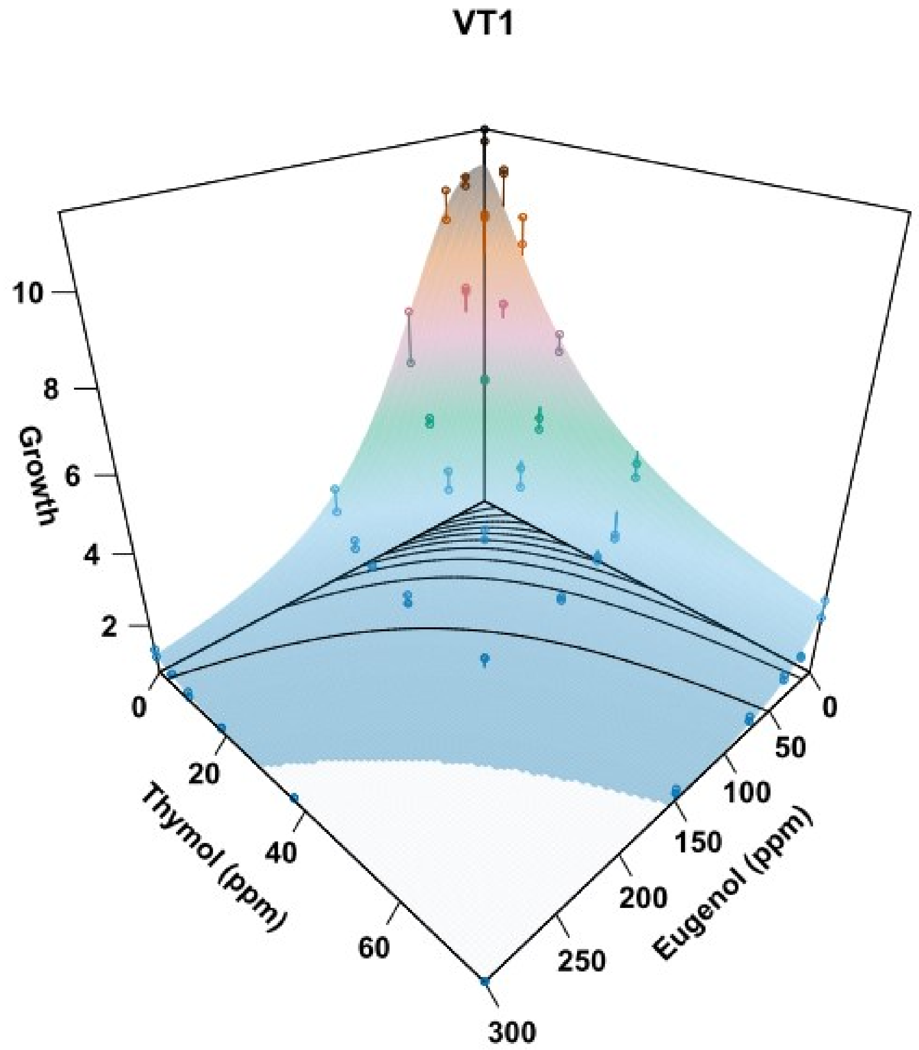

57 **Figure S9. Strain C1.1**

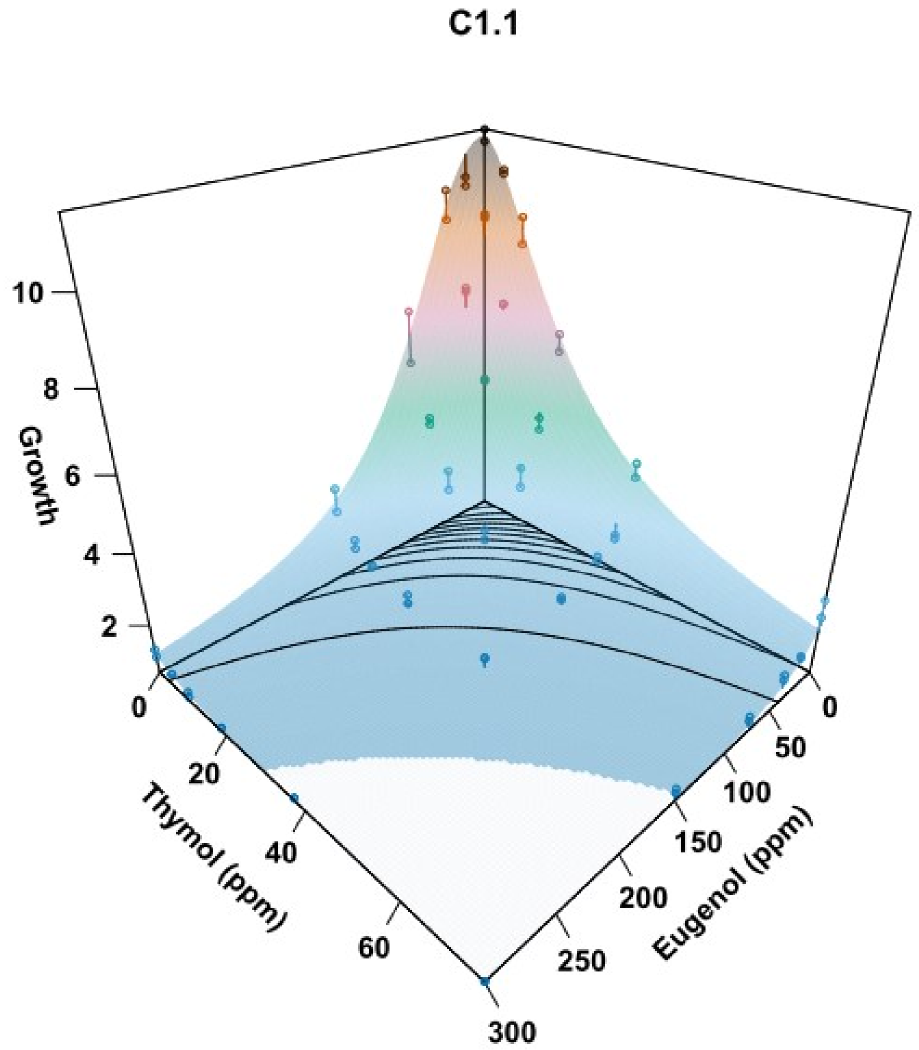

58

Figure S10. Strain S08

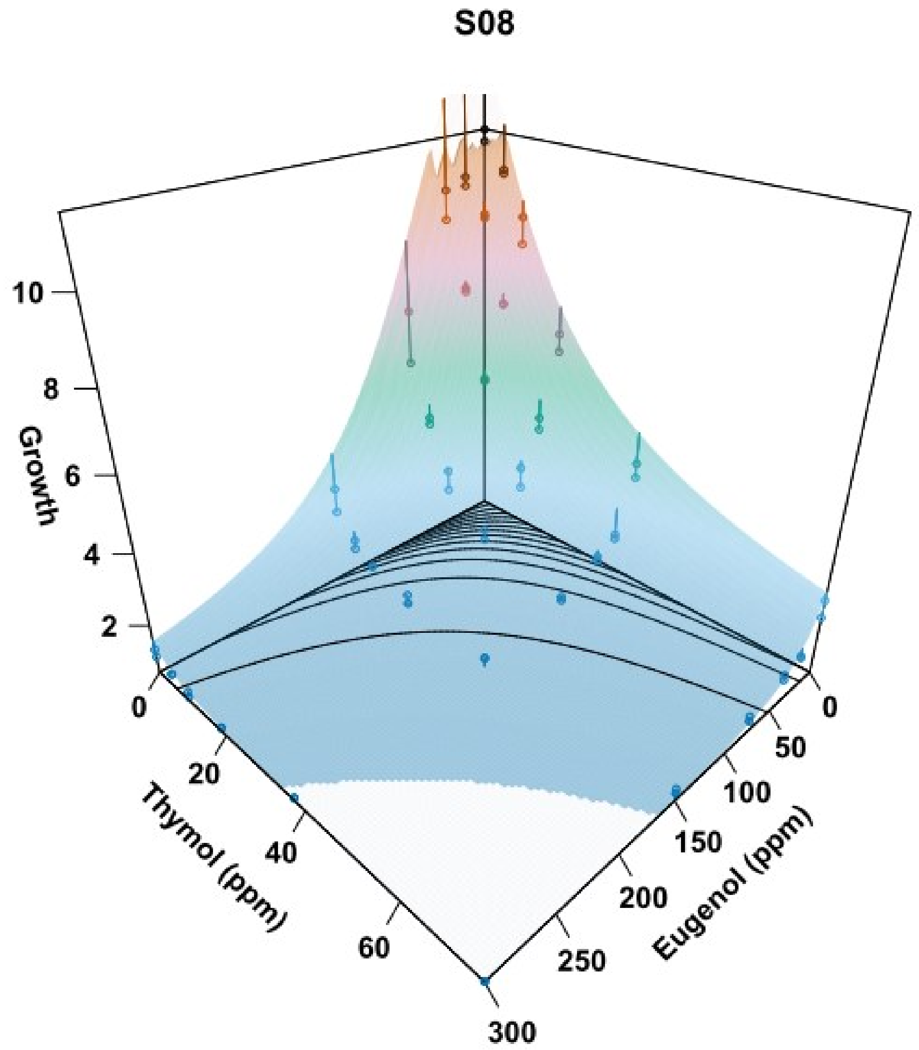

Supplement: Supplementary file 1 [file ECE3-7-1836-s001.pdf]
